# Navigating Public Microarray Databases

**DOI:** 10.1002/cfg.427

**Published:** 2004

**Authors:** Christopher J. Penkett, Jürg Bähler

**Affiliations:** The Wellcome Trust Sanger Institute Wellcome Trust Genome Campus Hinxton, Cambridge CB10 1SA UK

## Abstract

With the ever-escalating amount of data being produced by genome-wide microarray studies, it is of increasing importance that these data are captured in public databases so that researchers can use this information to complement and enhance their own studies. Many groups have set up databases of expression data, ranging from large repositories, which are designed to comprehensively capture all published data, through to more specialized databases. The public repositories, such as ArrayExpress at the European Bioinformatics Institute contain complete datasets in raw format in addition to processed data, whilst the specialist databases tend to provide downstream analysis of normalized data from more focused studies and data sources. Here we provide a guide to the use of these public microarray resources.


**Comparative and Functional Genomics**
*Comp Funct Genom* 2004; **5**: 471–479.
Published online in Wiley InterScience (www.interscience.wiley.com). **DOI:** 10.1002/cfg.427

**Review**

### Navigating public microarray databases

Christopher J. Penkett* and Jürg Bähler
*The Wellcome Trust Sanger Institute, Wellcome Trust Genome Campus, Hinxton, Cambridge CB10 1SA, UK*

**Correspondence to: Christopher J. Penkett, The Wellcome Trust Sanger Institute, Wellcome Trust Genome Campus, Hinxton, Cambridge, CB10 1SA, UK. E-mail: cjp@sanger.ac.uk*

Received: 25 June 2004
Revised: 12 August 2004
Accepted: 12 August 2004

### Abstract

**With the ever-escalating amount of data being produced by genome-wide microarray studies, it is of increasing importance that these data are captured in public databases so that researchers can use this information to complement and enhance their own studies. Many groups have set up databases of expression data, ranging from large repositories, which are designed to comprehensively capture all published data, through to more specialized databases. The public repositories, such as ArrayExpress at the European Bioinformatics Institute contain complete datasets in raw format in addition to processed data, whilst the specialist databases tend to provide downstream analysis of normalized data from more focused studies and data sources. Here we provide a guide to the use of these public microarray resources. Copyright © 2004 John Wiley & Sons, Ltd.**

**Keywords:** **microarray data; gene expression data; databases; public repositories; MIAME; MGED**

### Introduction

Microarrays have rapidly become the tool of choice for monitoring genome-wide levels of cellular gene expression [10,14,37,38]. The main reasons for this growth in usage are as follows: (a) as a result of the many genome- and EST-sequencing projects, there have been large increases in the number of DNA sequences that require functional information [39]; (b) the technology has become more accessible, allowing many groups to harness its power, in particular (i) the technology has improved over time and become easier to use, and (ii) it is less expensive to run; (c) finally, the informatics infrastructure, through both hardware and software improvements, has advanced quickly enough to keep pace with these new challenges in molecular biology [16].

There are two major types of informatics solutions that are required for the management of microarray data: (a) software, usually installed locally, that allows storage, querying and analysis of data captured on-site [2,17,19]; and (b) databases that act as repositories for publicly available data and, in particular, published data [2,9,18,19,25,30,35,40]. In this article, we shall briefly address the local packages by giving a list of the main ones that are currently available, and then we shall concentrate on the latter databases by giving details about how to use them and what data they contain.

### Local packages

In the late 1990s, as a rising number of both academic and commercial groups began to use commercial or home-made microarray platforms in earnest, a number of small bioinformatics companies started to develop software packages to capture and analyse the data being collected. One of the first companies in this field was Silicon Genetics, who developed the GeneSpring expression analysis software. This package took off rapidly as it was easy to use and contained most of the simple analysis features that users required.

Additional academic and commercial software packages that are currently available for microarray

Copyright © 2004 John Wiley & Sons, Ltd.

analysis are shown in Table 1. Packages that are principally available as web tools or desktop applications are shown in Section 1 of the table, while Section 2 shows software that also possess databases for the storage of microarray data (reviewed in [2,17]). In addition, statistical software can be tailored for the analysis of microarray data (listed in Section 3 of Table 1); e.g. there is the BioConductor package, which is a suite of tools for use with the freely available R package [17,47]. Links to many of these packages and other tools are provided at the following websites: **http://genex.sourceforge.net/other-tools.html**; **http://www.ifom-firc.it/MICRO-ARRAY/data_analysis.htm**; and **http://microarray.ccgb.umn.edu/smd/html/MicroArray/SMD/restech.html**

### Public repositories

The larger microarray groups tended to develop databases and software for the storage and analysis of their own data [1,2,13,17,19,20,22,27]. When data were published by these groups, they were made publicly available in different formats, usually over the web. With an ever-increasing amount of microarray data being published, it became clear that there was a need to store these data in central repositories, as had been the case in the sequencing and protein structure fields in previous decades.

Public repositories were initiated by the organizations that had previously collaborated to create the main sequence databases (see Section 1 of Table 2), viz. the NCBI (National Center for Biotechnology Information) in the US with its GEO database [18] (Gene Expression Omnibus), the EBI (European Bioinformatics Institute) in Europe with ArrayExpress [9,35], and the NIG (National Institute of Genetics) in Japan with CIBEX [25] (Center for Information Biology Gene EXpression database).

The aim of these repositories is to store the Minimum Information About a Microarray Experiment' (MIAME) [8], to allow researchers to replicate experiments. This has been developed by the international Microarray Gene Expression Data Society [5,6,31] (MGED Society; **http://www.mged.org/**). To the investigator, MIAME represents a checklist of information to be supplied during the experiment submission process. Initially, there had been a hope to save the raw array images centrally, but the acceleration in the amount of data collected meant that this was not going to be feasible in the longer term. Instead, all raw data from the files outputted by the image analysis programs are archived in these repositories. Additionally, a large amount of information is stored about the microarrays used in the experiments and the way they were produced, how the samples were obtained, protocols for RNA extraction and labelling, as well as methods used for sample hybridization, slide scanning and data normalization.

As researchers have become increasingly bold in utilizing microarray technologies, a variety of different experimental methods have evolved in addition to the usual transcription profiling technique; notably, these include comparative genomic hybridization (CGH [29]), chromatin-immunoprecipitation combined with arrays (ChIP-chip [33]), and both toxico- and nutri-genomics (**http://www.ebi.ac.uk/microarray/Projects/toxnutri/index.html**) methods. In addition, data are collected for a variety of organisms using different expression platforms. The databases need to be sufficiently flexible to cope with these variables, so the database groups have come up with different approaches to overcome these challenges [40]; e.g. GEO was primarily designed to act simply as a repository for public data, whilst an aim of ArrayExpress was to query and download datasets, and compare data from different experiments. In addition, the GEO database is flexible enough to store a variety of other high-throughput experimental data including serial analysis of gene expression (SAGE) and proteomics data [32].

Following the model of the sequence databases, the three international microarray database groups intend to exchange and share their data [9,25,35,40]. This has turned out to be much more difficult to achieve than for sequence data, due to the increased complexity of the data. The method that the groups have developed, in common with the way many informatics groups share data, is to use a common mark-up language called MicroArray Gene Expression Markup Language (MAGE-ML [41]), which is derived from the more general XML format. This means that any group using a database that can export in MAGE-ML format should be able to transfer their data, with relative ease, into one of the central repositories.

Copyright © 2004 John Wiley & Sons, Ltd. 

**Table 1.** Commercial and academic software packages available for microarray data analysis

| Application | Organization | URL |
|---|---|---|
| 1. Desktop and web applications | | |
| ArrayMiner | Optimal Design | **http://www.optimaldesign.com/ArrayMiner/ArrayMiner.htm** |
| ArrayStat | Imaging Research | **http://www.imagingresearch.com/products/AST.asp** |
| BRB ArrayTools | National Cancer Institute (NCI) | **http://linus.nci.nih.gov/BRB-ArrayTools.html** |
| Cyber-T | University of California, Irvine | **http://visitor.ics.uci.edu/genex/cybert/** |
| Decision Site for Functional Genomics | SpotFire | **http://www.spotfire.com/academics/products-fg.htm** |
| Expression Profiler | European Bioinformatics Institute (EBI) | **http://ep.ebi.ac.uk/EP/** |
| GAAS (Gene Array Analyser Software) | Politecnico di Milano | **http://www.medinfopoli.polimi.it/GAAS/** |
| GCOS (GeneChip Operating Software) | Affymetrix | **http://www.affymetrix.com/products/software/index.affx** |
| GeneMaths XT | Applied Maths | **http://www.applied-maths.com/genemaths/genemaths.htm** |
| GeneSight | BioDiscovery | **http://www.biodiscovery.com/genesight.asp** |
| GeneSpring | Silicon Genetics | **http://www.silicongenetics.com/cgi/SiG.cgi/Products/GeneSpring/index.smf** |
| Genesis | Graz University | **http://genome.tugraz.at/Software/Genesis/Genesis.html** |
| J-Express Pro | MolMine | **http://www.molmine.com/frameset/frm_jexpress.htm** |
| Vector Xpression | Informax | **http://register.informaxinc.com/solutions/xpression/main.html** |
| 2. Database packages | | |
| Acuity | Axon Instruments | **http://www.axon.com/gn_Acuity.html** |
| BASE (BioArray Software Environment) | Lund University | **http://base.thep.lu.se/index.phtml** |
| Expressionist Pro | GeneData | **http://www.genedata.com/new_products_template.php?basename=expressionist_pro** |
| GeneDirector | BioDiscovery | **http://www.biodiscovery.com/genedirector.asp** |
| GeNet | Silicon Genetics | **http://www.silicongenetics.com/cgi/SiG.cgi/Products/GeNet/index.smf** |
| GeneTraffic | Iobion Informatics LLC | **http://www.iobion.com/products/products.html** |
| GeneX-Lite | National Center for Genome Research (NCGR) | **http://www.ncgr.org/genex/** |
| LAD (Longhorn Array Database) | University of Texas, Austin | **http://www.longhornarraydatabase.org/** |
| maxd | University of Manchester | **http://bioinf.man.ac.uk/microarray/maxd/** |
| PARTISAN arrayLIMS | Clondiag | **http://www.clondiag.com/products/sw/partisan/index.php** |
| Resolver | Rosetta Inpharmatics, Inc. | **http://www.rosettabio.com/products/resolver/default.htm** |
| TM4 (Microarray Software Suite) | The Institute for Genomic Research (TIGR) | **http://www.tigr.org/software/tm4/** |
| 3. Statistical packages | | |
| The R Package (BioConductor) | Bell Laboratories | **http://cran.r-project.org/and http://www.bioconductor.org/** |
| MatLab (Bioinformatics Toolbox) | The MathWorks | **http://www.mathworks.com/products/bioinfo/** |
| Partek Pro | Partek | **http://www.partek.com/html/products/products.html** |
| S+ (ArrayAnalyzer) | Insightful | **http://www.insightful.com/products/s-plus_arrayanalyzer/default.asp** |
| SAS (SAS Microarray) | SAS | **http://www.sas.com/industry/pharma/mas/index.html** |

Copyright © 2004 John Wiley & Sons, Ltd.

**Table 2.** Databases containing public microarray datasets

| Database name | Organization | URL | Notes |
|---|---|---|---|
| 1. International repositories | | | |
| ArrayExpress [9,35] | European Bioinformatics Institute (EBI) | **http://www.ebi.ac.uk/arrayexpress/** | European MIAME-compliant database of published data; can use MIAMExpress web tool to submit data |
| CIBEX [25] (Center for Information Biology Gene Expression database) | Center for Information Biology (CIB), National Institute for Genetics (NIG) | **http://cibex.nig.ac.jp/** | Japanese public database with search and submission capabilities; can view data in a graphical array-like format |
| GEO [18] (Gene Expression Omnibus) | National Center for Biotechnology Information (NCBI) National Institutes of Health (NIH) | **http://www.ncbi.nih.gov/geo/** | US public database; has browsing, searching and submission functions similar in appearance to Entrez |
| 2. Other general expression databases | | | |
| CleanEx [34] | Swiss Institute of Bioinformatics (SIB) and Swiss Institute for Experimental Cancer Research (ISREC) | **http://www.cleanex.isb-sib.ch/** | Curated database for array data collected using many technologies; the expression data is linked to genes using up-to-date consistent nomenclature |
| RAD [43] (RNA Abundance Database) | University of Pennsylvania | **http://www.cbil.upenn.edu/RAD/** | Schema based on MAGE and MIAME; data can be viewed with an MA graphical plot |
| SMD [20] (Stanford Microarray Database) | Stanford University | **http://genome-www.stanford.edu/microarray/** | Data for Stanford investigators and their collaborators; very comprehensive database that allows a wide range of analysis and plotting features, including clickable raw images |
| 3. Project-specific databases | | | |
| EPConDB [26] (Endocrine Pancreas Consortium Database) | University of Pennsylvania | **http://www.cbil.upenn.edu/EPConDB/** | Mainly a database of annotations with regard to pancreatic sequences in human and mouse; contains expression data for three mouse studies; easier to download data than to query it |
| GermOnline [46] | International Consortium | **http://www.germonline.org/** | Data regarding germ cell growth and development in 11 model organisms, including yeasts, plants, *C. elegans, Drosophila*, vertebrates, mammals and human; relevant genes have links to graphs of expression data both locally and at other web sites |
| HPMR [7] (Human Plasma Membrane Receptome) | Stanford University | **http://receptome.stanford.edu/** | Information about human plasma membrane receptors; can search for receptor genes and shows data in the Gene Expression Atlas database |
| PEPR [12] (Public Expression Profiling Resource) | Children's National Medical Center and George Washington University | **http://microarray.cnmcresearch.org/** | Focuses on lung diseases using human, mouse and rat Affymetrix data; in most cases, it is possible only to download the data for different experiments |

Copyright © 2004 John Wiley & Sons, Ltd. 

| | | | |
|---|---|---|---|
| 4. Human/mouse tissue databases | | | |
| Gene Expression Atlas [44,45] | Genomics Institute of the Novartis Research Foundation | **http://symatlas.gnf.org/SymAtlas/** | Affymetrix data for human and mouse tissues and cell lines with various standard and custom chips; genes can be searched by annotation or expression levels; for each matching gene, a bar/pie chart of expression levels in the different cells is shown alongside gene annotation; can also search for genes with similar tissue expression profiles |
| GeneNote [11] | Weizmann Institute of Science | **http://genecards.weizmann.ac.il/genenote/** | Affymetrix data for 12 healthy human tissues (U95A-E); also shows SAGE and electronic Northern unique clone counts; linked to well-annotated GeneCards database |
| HugeIndex [23] | Boston University and Harvard University | **http://www.hugeindex.org/** | Affymetrix data for 19 normal human tissues (Hu6800); can search for gene expression patterns in these tissues, genes that are present or absent in various combinations of the tissues, and do scatter plots of data between different tissues |
| READ [21] (RIKEN cDNA Expression Array Database) | RIKEN Yokohama Institute | **http://read.gsc.riken.go.jp/** | cDNA data for 49 adult and embryonic mouse tissues using RIKEN clones; can search for individual clones or high/low expressers in combinations of tissues; genes with similar tissue patterns can also be found |
| RefExA (Reference Database for Gene Expression Analysis) | The University of Tokyo | **http://www.lsbm.org/site_e/index.html** | Affymetrix data for 80 normal and cancerous human tissues (U133); genes can be searched and viewed by using a wide variety of annotation information; expression data is shown as colour-coded block or bar charts; some data can also be viewed using a GeNet server |
| 5. Organism-specific databases | | | |
| *Sz. pombe* GeneDB [24] | The Wellcome Trust Sanger Institute | **http://www.genedb.org/genedb/pombe/index.jsp** | Mainly a gene annotation database; includes plots of data for cell cycle, meiosis and stress response for individual *Sz. pombe* genes |
| NASCArrays [15] (Nottingham *Arabidopsis* Stock Centre Arrays database) | University of Nottingham | **http://affymetrix.arabidopsis.info/** | Allows downloading, plotting and clustering of *Arabidopsis* Affymetrix data; experimental descriptions are MIAME-compliant |
| PlasmoDB [3] (*Plasmodium* Genome Database) | International Consortium | **http://www.plasmodb.org/** | Mainly a gene annotation database; includes time series data for life cycle of *Plasmodium*, which can be visualized with plots; genes with similar profiles to a gene of interest can also be searched for |
| SGD (*Saccharomyces* Genome Database) Expression Connection [4] | Stanford University | **http://genome-www4.stanford.edu/cgi-bin/SGD/expression/expressionConnection.pl** | Expression data in SGD; data from 16 different studies with *S. cerevisiae* can be searched, filtered and plotted |
| yMGV [28] (Yeast Microarray Global Viewer) | École Normale Supérieure | **http://www.transcriptome.ens.fr/ymgv/** | Data for 82 *S. cerevisiae* and two *Sz. pombe* studies; individual genes can be searched for and data can be visualized graphically; genes that vary most and least often in each species can also be searched for |

Copyright © 2004 John Wiley & Sons, Ltd.

The use of controlled nomenclature or ontologies can also ease the data-sharing process. A working group has been set up to standardize common terms and phrases [42] (see **http://mged.sourceforge.net/ontologies/index.php**); this will probably remain an ongoing process as the technology develops and finds new applications.

### Data submission to public repositories

Currently, the major issue for public repositories is the submission process, because of the variation between datasets from different groups and studies. For example, experimental designs vary widely: some researchers do time courses, perhaps with a pooled reference sample, whereas others do studies comparing normal against mutant or diseased cells/tissues. There are a wide variety of microarray platforms, all of which need to be described in detail. Furthermore, some of these technologies record different types of data: an Affymetrix GeneChip records one sample per chip and has several perfect match and mismatch probes per gene, whilst a two-colour microarray captures competitive hybridization between two samples, often with multiple replicates for many genes. Inevitably, the complexity of all these features makes a system that attempts to facilitate the conversion of this information into MAGE-ML somewhat unwieldy.

A few organizations have developed their own pipelines for the creation of MAGE-ML, which allow them to submit data in a more automated fashion [6,9,35]. Hopefully, as MAGE-ML and the ontologies mature, software manufacturers will develop user interfaces that permit an easier and more uniform submission process. The ArrayExpress group have been developing a web application, MIAMExpress, which allows researchers to submit their data to ArrayExpress. The submission process has been designed by a team of software developers working closely with data curators [9,35]. This software allows for the importing of experiments of more than 100 arrays into ArrayExpress. Initially, it is possible to keep the data private, and the investigator can specify when to make the data publicly available. This means that data can be submitted as they are collected, so that the system can trace experiments electronically and independently of a lab book. However, in our experience, except for smaller datasets, it is beneficial to have a bit of experience of the MIAMExpress front-end when submitting data through it, e.g. it is useful to have an understanding of the whole submission process to keep track of where you are in the process.

Since the release of MAGE-ML, other academic groups have been working to establish fully working MAGE-ML-based pipelines for importing data automatically into ArrayExpress from their databases; these databases include: Stanford Microarray Database, Stanford University (SMD [20]); RNA Abundance Database, University of Pennsylvania (RAD [43]); the TM4 Microarray Software Suite from The Institute for Genomic Research (TIGR [36]); and the microarray database at the German Resource Center for Genome Research (RZPD), **http://www.rzpd.de/submit/**

At the Sanger Institute, Matloob Qureshi has been working on a user-friendly Java application that will allow members of both the Pathogen Microarray Group and our group to submit their data in MAGE-ML format to ArrayExpress. The advantage with this type of software is that with the increased flexibility of Java applications compared to web applications, it is easier to have an overview of the submission process. The main reason that the ArrayExpress team adopted a web application is that this method supports data submission from remote laboratories with little local computing support [35]. Hence, for those with good local IT support, tools that make the process easier and more automated will become of increasing value if they wish to submit large amounts of data to ArrayExpress or the other central repositories.

### Accessing data from public repositories

The value of the central resources (ArrayExpress, GEO and CIBEX) will increase as more datasets are submitted to them. Prior to the establishment of these repositories, data were not always or continuously available, were hard to find on the Internet, and were stored in varying formats. Clearly, databases with professional support that provide standardized datasets and stable URL addresses are essential for the long-term benefit of obtaining and re-analysing data from published microarray studies. There are now a variety of datasets that are available at ArrayExpress, including data

Copyright © 2004 John Wiley & Sons, Ltd. 

for humans, human cell lines, rodents, plants, yeast and bacteria; this number is set to increase rapidly, e.g. in February 2004 there were only four datasets for the yeast *Saccharomyces cerevisiae*, but 5 months later the number of datasets stands at 16.

An ever-changing aspect of microarray data is the sequence annotation on the arrays, which is being constantly updated and improved. ArrayExpress solves this problem by linking from the sequences on each array to external annotation databases, e.g. we include links to *Schizosaccharomyces pombe* GeneDB [24] (a public database for fission yeast genes: **http://www.genedb.org/**) when we submit a newly designed *Sz. pombe* array to the database in array description file (ADF) format.

### Specialized public databases

Public datasets are stored in a number of other databases besides the public repositories. The less stringent requirements of those databases that do not use the MIAME checklist present a smaller submission burden for researchers, hence they often contain more datasets than the main repositories. The nature of these databases means they have differing priorities and structures (Table 2; and [30]), e.g. some databases contain large amounts of publicly available data, but are only open to submission by a defined group of researchers (Section 2 of Table 2); SMD is perhaps the most established of these and includes many valuable analysis features [20].

Other databases relate to specific projects or biological processes (Section 3 of Table 2), e.g. GermOnline contains expression data relevant to the mitotic and meiotic cell cycle in both yeast and higher eukaryotes [46]. Additionally, there are a number of databases that store tissue distribution information for human and mouse cell lines (Section 4 of Table 2). One easily searchable example is the Gene Expression Atlas database, which contains Affymetrix profiles for 79 human and 61 mouse tissues collected for a variety of up-to-date standard and custom chips [44,45]; it is possible to search for genes with similar expression profiles and genes include extensive annotation alongside the expression data. Finally, some databases store data for related organisms (Section 5 of Table 2); an example is the yeast microarray database, yMGV [28]. This database provides useful tables and graphs of datasets from different yeast species.

Lists of such specialized databases are available from: **http://www3.oup.co.uk/nar/database/cat/9** and **http://ihome.cuhk.edu.hk/b400559/array-soft_public.html**. However, it is apparent that some older sites have broken links or are out-of-date, leading to the loss of access, further demonstrating the importance of maintaining central repositories.

### Conclusions

Since the launch of the central microarray repositories, the submission procedures for these resources have been made easier and more accessible. The value of centralized warehouses that contain standardized, well-structured data is clear, and has been established for some time now in other fields. Although not yet straightforward, the time is fast approaching when all researchers should submit their data to public repositories so that all expression data are kept together, as the current open letter from the MGED society suggests [6]. See also the recent article by Ball *et al.* [48].

It is also valuable to have specialized databases of microarray data, which are available through the web and designed with expert local biological knowledge, as these databases will allow researchers to focus on the data that are most relevant to them. Such resources will also benefit from being able to obtain all the data they require from the central public repositories.

### Acknowledgements

The authors wish to thank Alvis Brazma for valuable additions to the paper. We also thank Matloob Qureshi for supplying the script that creates ADF files for our arrays, and Rob Andrews, Philippe Rocca-Serra and Helen Parkinson for their help with our data submissions to ArrayExpress. The work in our laboratory is funded by Cancer Research UK [CUK], Grant No. C9546/A5262.

### References

1. Aach J, Rindone W, Church GM. 2000. Systematic management and analysis of yeast gene expression data. *Genome Res* **10**: 431–445.

Copyright © 2004 John Wiley & Sons, Ltd.

2. Anderle P, Duval M, Draghici S, *et al*. 2003. Gene expression databases and data mining. *Biotechniques Suppl* **Mar**: 36–44.
3. Bahl A, Brunk B, Crabtree J, *et al*. 2003. PlasmoDB: the *Plasmodium* genome resource. A database integrating experimental and computational data. *Nucleic Acids Res* **31**: 212–215.
4. Ball CA, Jin H, Sherlock G, *et al*. 2001. *Saccharomyces* Genome Database provides tools to survey gene expression and functional analysis data. *Nucleic Acids Res* **29**: 80–81.
5. Ball CA, Sherlock G, Parkinson H, *et al*. 2002. A guide to microarray experiments — an open letter to the scientific journals. *Lancet* **360**: 1019.
6. Ball CA, Brazma A, Causton H, *et al*. 2004. Submission of microarray data to public repositories. *PLoS Biol* **2**: E317.
7. Ben-Shlomo I, Yu Hsu S, Rauch R, Kowalski HW, Hsueh AJ. 2003. Signaling receptome: a genomic and evolutionary perspective of plasma membrane receptors involved in signal transduction. *Sci STKE* **187**: RE9.
8. Brazma A, Hingamp P, Quackenbush J, *et al*. 2001. Minimum information about a microarray experiment (MIAME) — toward standards for microarray data. *Nature Genet* **29**: 365–371.
9. Brazma A, Parkinson H, Sarkans U, *et al*. 2003. ArrayExpress — a public repository for microarray gene expression data at the EBI. *Nucleic Acids Res* **31**: 68–71.
10. Brazma A, Robinson A, Cameron G, Ashburner M. 2000. One-stop shop for microarray data. *Nature* **403**: 699–700.
11. Chalifa-Caspi V, Shmueli O, Benjamin-Rodrig H, *et al*. 2003. GeneAnnot: interfacing GeneCards with high-throughput gene expression compendia. *Brief Bioinform* **4**: 349–360.
12. Chen J, Zhao P, Massaro D, *et al*. 2004. The PEPR GeneChip data warehouse, and implementation of a dynamic time series query tool (SGQT) with graphical interface. *Nucleic Acids Res* **32**: D578–581.
13. Cheung KH, White K, Hager J, *et al*. 2002. YMD: a microarray database for large-scale gene expression analysis. *Proc AMIA Sympos*, 140–144.
14. Chipping Forecast II. 2002. *Nature Genet* **32**: (suppl): 461–552.
15. Craigon DJ, James N, Okyere J, *et al*. 2004. NASCArrays: a repository for microarray data generated by NASC's transcriptomics service. *Nucleic Acids Res* **32**: D575–577.
16. Dennis C. 2002. Information overload. *Nature* **417**: 14.
17. Dudoit S, Gentleman RC, Quackenbush J. 2003. Open source software for the analysis of microarray data. *Biotechniques Suppl* **Mar**: 45–51.
18. Edgar R, Domrachev M, Lash AE. 2002. Gene Expression Omnibus: NCBI gene expression and hybridization array data repository. *Nucleic Acids Res* **30**: 207–210.
19. Gardiner-Garden M, Littlejohn TG. 2001. A comparison of microarray databases. *Brief Bioinform* **2**: 143–158.
20. Gollub J, Ball CA, Binkley G, *et al*. 2003. The Stanford Microarray Database: data access and quality assessment tools. *Nucleic Acids Res* **31**: 94–96.
21. Grimmond SM, Miranda KC, Yuan Z, *et al*. 2003. The mouse secretome: functional classification of the proteins secreted into the extracellular environment. *Genome Res* **13**: 1350–1359.
22. Harrison R, DeLisi C. 2002. Condition specific transcription factor binding site characterization in *Saccharomyces cerevisiae*. *Bioinformatics* **18**: 1289–1296.
23. Haverty PM, Weng Z, Best NL, *et al*. 2002. HugeIndex: a database with visualization tools for high-density oligonucleotide array data from normal human tissues. *Nucleic Acids Res* **30**: 214–217.
24. Hertz-Fowler C, Peacock CS, Wood V, *et al*. 2004. GeneDB: a resource for prokaryotic and eukaryotic organisms. *Nucleic Acids Res* **32**: D339–343.
25. Ikeo K, Ishi-i J, Tamura T, Gojobori T, Tateno Y. 2003. CIBEX: Center for Information Biology gene expression database. *Comptes Rendus Biol* **326**: 1079–1082.
26. Kaestner KH, Lee CS, Scearce LM, *et al*. 2003. Transcriptional program of the endocrine pancreas in mice and humans. *Diabetes* **52**: 1604–1610.
27. Killion PJ, Sherlock G, Iyer VR. 2003. The Longhorn Array Database (LAD): an open-source, MIAME compliant implementation of the Stanford Microarray Database (SMD). *BMC Bioinformat* **4**: 32.
28. Lelandais G, Le Crom S, Devaux F, *et al*. 2004. yMGV: a cross-species expression data mining tool. *Nucleic Acids Res* **32**: D323–325.
29. Mantripragada KK, Buckley PG, de Stahl TD, Dumanski JP. 2004. Genomic microarrays in the spotlight. *Trends Genet* **20**: 87–94.
30. Moreau Y, Aerts S, De Moor B, De Strooper B, Dabrowski M. 2003. Comparison and meta-analysis of microarray data: from the bench to the computer desk. *Trends Genet* **19**: 570–577.
31. Nature Publishing Group. 2002. Microarray standards at last. *Nature* **419**: 323.
32. NCBI News. 2003. New data query and visualization tools for Gene Expression Omnibus (GEO). *NCBI News* **Summer**: 2.
33. Pollack JR, Iyer V. 2001. Characterizing the physical genome. *Nature Genet* **32**: (suppl): 515–521.
34. Praz V, Jagannathan V, Bucher P. 2004. CleanEx: a database of heterogeneous gene expression data based on a consistent gene nomenclature. *Nucleic Acids Res* **32**: D542–547.
35. Rocca-Serra P, Brazma A, Parkinson H, *et al*. 2003. ArrayExpress: a public database of gene expression data at EBI. *Comptes Rendus Biol* **326**: 1075–1078.
36. Saeed AI, Sharov V, White J, *et al*. 2003. TM4: a free, open-source system for microarray data management and analysis. *Biotechniques* **34**: 374–378.
37. Schena M, Shalon D, Davis RW, Brown PO. 1995. Quantitative monitoring of gene expression patterns with a complementary DNA microarray. *Science* **270**: 467–470.
38. Schulze A, Downward J. 2001. Navigating gene expression using microarrays — a technology review. *Nature Cell Biol* **3**: E190–195.
39. Searls DB. 2003. Bioinformatics tools for whole genomes. *Annu Rev Genom Hum Genet* **1**: 251–279.
40. Spellman PT. 2001. The future of publishing microarray data. *Brief Bioinform* **2**: 316–318.
41. Spellman PT, Miller M, Stewart J, *et al*. 2002. Design and implementation of microarray gene expression markup language (MAGE-ML). *Genome Biol* **3**: research 0046.1–0046.9.
42. Stoeckert CJ Jr, Causton HC, Ball CA. 2002. Microarray databases: standards and ontologies. *Nature Genet* **32**: (suppl): 469–473.
43. Stoeckert C, Pizarro A, Manduchi E, *et al*. 2001. A relational schema for both array-based and SAGE gene expression experiments. *Bioinformatics* **17**: 300–308.

Copyright © 2004 John Wiley & Sons, Ltd. 

44. Su AI, Cooke MP, Ching KA, *et al*. 2002. Large-scale analysis of the human and mouse transcriptomes. *Proc Natl Acad Sci USA* **99**: 4465–4470.
45. Su AI, Wiltshire T, Batalov S, *et al*. 2004. A gene atlas of the mouse and human protein-encoding transcriptomes. *Proc Natl Acad Sci USA* **101**: 6062–6067.
46. Wiederkehr C, Basavaraj R, Sarrauste de Menthiere C, *et al*. 2004. GermOnline, a cross-species community knowledge base on germ cell differentiation. *Nucleic Acids Res* **32**: D560–567.
47. Gentleman RC, Carey VJ, Bates DM, *et al*. 2004. Bioconductor: open software development for computational biology and bioinformatics. *Genome Biol* **5**: R80.
48. Ball CA, Sherlock G, Brazma A. 2004. Funding high-throughput data sharing. *Nat Biotech* **22**: 1179–1183.

Copyright © 2004 John Wiley & Sons, Ltd. 
